# Sensitivity of Enzyme-Linked Immunosorbent Assay in Monitoring Treatment Response in Phospholipase A2 Receptor –Associated Membranous Nephropathy

**DOI:** 10.1016/j.ekir.2025.07.043

**Published:** 2025-08-06

**Authors:** Coralien Vink, Ruben Visch, Jack Wetzels, Anne-Els van de Logt

**Affiliations:** 1 Department of Nephrology, Radboudumc Institute for Medical Innovation, Radboud University Medical Center, Nijmegen, The Netherlands

**Keywords:** enzyme-linked immunosorbent assay, immunofluorescence test, membranous nephropathy, M-type phospholipase A2 receptor

## Introduction

The identification in 2009 of phospholipase A2 receptor (PLA2R) 1 as major auto-antigen in membranous nephropathy has been instrumental in improving patient management.^1^ Historically, the first commercially available test to detect antibodies against PLA2R 1 (aPLA2Rab) was the immunofluorescence test (IFT).^2^ This assay proved sensitive, but lacks accuracy in quantifying aPLA2Rab levels. The introduction of the enzyme-linked immunosorbent assay (ELISA) allowed quantification, with levels expressed in arbitrary units. A cut-off value of 14 RU/ml or 20 RU/ml was proposed to distinguish between aPLAR2Rab negativity and aPLA2Rab positivity. Importantly, this cutoff was developed for diagnostic purposes, thus aiming to be specific (few false positives). For this goal, the ELISA test proved slightly less sensitive than the IFT test (reported at 96.5%).^3^ Although the ELISA test is now often used, the sensitivity of the test in monitoring treatment response is unknown. In some studies, the cut-off level of 14 RU/ml is used to define immunological remission. In contrast, the Kidney Disease: Improving Global Outcomes guideline suggests to use a cutoff of 2 RU/ml.^4^ This suggestion was based on expert opinion, in the absence of clinical study data.

Immunological remission, defined as disappearance of aPLA2Rab, is considered the primary goal of therapy in membranous nephropathy and it is expected that monitoring the course of aPLA2Rab after start of therapy may aid in adapting dose, duration, and type of therapy. Indeed, we recently reported the results of a prospective study, in which cyclophosphamide-based therapy was guided by aPLA2Rab response.^5^ In this study, treatment was stopped at the time of immunological remission, defined as disappearance of aPLA2Rab measured by IFT. In many patients, treatment duration could be limited to 8 weeks.

In the present study, we studied the sensitivity of ELISA compared with IFT during follow-up after start of therapy in 2 distinct cohorts as follows: (i) a prospective analysis of samples obtained in patients who participated in our study, with samples stored at baseline and after 8 weeks of CP therapy^5^; and (ii) a retrospective analysis of parallel performed ELISA and IFT tests in samples taken in patients with membranous nephropathy with data available in our research database. (Supplementary Methods).

## Results

### Prospective Cohort

Our study included 50 incident patients, and baseline samples were available in 46 patients (43 Caucasian, 5 diabetes) and samples obtained after 8 weeks of therapy were available in 38 patients. Using baseline samples, and applying the cutoff of 14 RU/ml, the ELISA assay had a sensitivity of 98% (Table 1). A cutoff of 20 RU/ml was slightly less sensitive (96 %). In contrast, using samples obtained during therapy, the sensitivity of the ELISA was much lower at 31% and 62% when applying a cut-off value of 20 RU/ml and 14 RU/ml, respectively (Table 1). The clinical consequences can be described as follows: there were 13 patients with available samples and positive IFT results after 8 weeks, in whom per-protocol treatment was continued. Of these patients, aPLA2Rab levels were < 20 RU/ml in 9 and < 14 RU/ml in 5 (ELISA). Using ELISA and the manufacturer-recommended cut-off values would have resulted in earlier withdrawal of therapy (see discussion). Of note, all samples with ELISA < 2 RU/ml proved IFT negative.

**Table 1:** Agreement of aPLA2Rab results measured with IFT and ELISA technique in samples collected before and after 8 weeks of immunosuppressive therapy

Baseline samples					
ELISA titer	< 2	2–14	14–20	> 20	Total
IFT positive	0	1	1	44	46
8 weeks samples					
IFT negative	16	9	0	0	25
IFT positive	0	5	4	4	13
Total 8 weeks samples	16	14	4	4	38

aPLA2Rab, anti–phospholipase A2 receptor antibody; ELISA, enzyme-linked immunosorbent assay; IFT, immunofluorescence test.

Calculated sensitivity/specificity:.

Diagnosis: cut-off 14 RU/ml sensitivity 98%.

Monitoring: cut-off 14 RU/ml sensitivity 62%, specificity 100%.

Monitoring cut-off 20 RU/ml sensitivity 31%, specificity 100%.

Monitoring cut-off 2RU/ml sensitivity 100%, specificity 64%.

### Retrospective Cohort

In the retrospective analysis of samples collected in a period of 12 months after start of therapy, we included 534 samples of 206 unique patients (Caucasian 95%, diabetes in 26 patients [13%]). In these samples, a cutoff of 14 RU/ml provided a sensitivity of only 47.6% (Table 2). A cutoff of 20 RU/ml was even less sensitive (38.7%). Of note, in this cohort, ELISA < 2 RU/ml did not provide 100% sensitivity (93%). There were 18 samples with ELISA < 2 RU/ml and positive IFT. These samples were from 12 unique patients. We evaluated these patients in more detail and observed that IFT became negative without additional therapy in 9 patients. Details of these “truly false negative” patients are provided in the Supplementary Material. For clinical use, the sensitivity of the ELISA using a cut-off value of < 2 RU/ml approaches 98%.

**Table 2:** Agreement of aPLA2Rab results measured with IFT and ELISA technique in samples collected within 12 months after the start of immunosuppressive therapy

Samples during 12 months of follow-up					
ELISA titer	< 2	2–14	14–20	> 20	Total
IFT negative	180	84	1	0	265
IFT positive	18	123	24	104	269

aPLA2Rab, anti–phospholipase A2 receptor antibody; ELISA, enzyme-linked immunosorbent assay; IFT, immunofluorescence test.

Calculated sensitivity/specificity:.

Monitoring: cut-off 14 RU/ml sensitivity 49%, specificity 100%.

Monitoring cut-off 20 RU/ml sensitivity 39%, specificity 100%.

Monitoring cut-off 2RU/ml sensitivity 93%, specificity 68%.

## Discussion

It is well-known that for diagnostic purposes, the ELISA test using a cutoff of 14 RU/ml is slightly less sensitive than with IFT. This cut-off value was based on test results in healthy and diseases controls, with a high value chosen to prevent too many false-positive results. Because immunological remission is an important goal of therapy, it is necessary to define cut-off values for monitoring purposes. Given that monitoring of PLA2Rab will only be relevant in patients with PLA2Rab-associated membranous nephropathy, false positivity is not a major issue.

Our prospective data clearly show that the sensitivity of ELISA is < 50% when using 14 RU/ml as a threshold to monitor response, whereas specificity is close to 100%. Obviously, it can be debated if, when monitoring treatment response we need to pursue a very high sensitivity or a very high specificity. Based on our prospective study, we argue that high sensitivity is needed. When using the cutoff of 14 RU/ml, therapy would have stopped earlier (Table 1, therapy would have stopped in 5 patients out of 13 with positive IFT). In our previous study, we concluded that withdrawal of therapy at the time point that IFT became negative was sometimes too early, because one-quarter of these patients needed a second course of therapy.^5^ ELISA levels < 2 RU/ml proved highly sensitive, although admittedly the number of samples was limited.

To validate our findings, we evaluated data of patients included in our retrospective database. We confirmed the lower sensitivity of the ELISA test using the cutoff levels of 14 or 20 RU/ml. Somewhat unexpectedly, the data suggested that even ELISA values < 2 RU/ml could prove false negative, because 9% (18/198) of tests remained IFT positive in 12 unique patients. However, in 9 of 12 of these patients, the IFT became negative without additional therapy in repeated tests; suggesting that the IFT in these patients may have allowed detection of very low, clinically irrelevant PLA2Rab levels. Therefore, the clinically relevant false negative rate is low at 2.5% of samples (5/198) and in 2.6% of patients (3/115) (Supplementary Material).

A limitation of our study is that approximately 95% of both patients are of Caucasian descent. Therefore, we cannot draw conclusions about the sensitivity of the ELISA in patients from different ethnicities. In addition, because few patients had diabetes and no patients had diabetic nephropathy, our conclusions might not apply to patients with diabetes.

In conclusion, our data indicate that for the assessment of immunological remission, the IFT test is most suited. When using the ELISA test, we suggest that a value < 2 RU/ml can be used to define immunological remission, whereas a value > 14 RU/ml reflects persistent immunological activity. We advise performing additional IFT tests in patients with ELISA values between 2 and 14 RU/ml.

## Disclosure

All the authors declared no competing interests.

## Supplementary Material

Supplementary File (PDF) **Supplementary Methods.** Summary of 3 patients with apparent false negative ELISA.
