# Supplementary material for: Sensitivity of Enzyme-Linked Immunosorbent Assay in Monitoring Treatment Response in Phospholipase A2 Receptor –Associated Membranous Nephropathy
Source: Kidney Int Rep. 2025 Aug 6;10(10):3661–3. doi: 10.1016/j.ekir.2025.07.043 (PMC12545688; doi:10.1016/j.ekir.2025.07.043)
Supplement: Supplementary File (PDF) — Supplementary Methods. Summary of 3 patients with apparent false negative ELISA. [file mmc1.pdf]

Supplementary Material: Sensitivity of ELISA in monitoring treatment response in PLA2R-associated membranous nephropathy

Supplementary Methods

Summary of three patients with apparent false negative ELISA (ELISA < 2 RU/ml and positive IFT)

Supplementary Methods

For the first part of this study stored serum samples obtained at baseline and after 8 weeks of therapy in incident patients participating in the above mentioned study were analyzed. Stored serum samples were retrieved and shipped to the Institute for Experimental Immunology, affiliated to EUROIMMUN Medizinische Labordiagnostika AG, Lübeck, Germany and analyzed by the commercial anti-PLA2R enzyme-linked immunosorbent assay (ELISA) from EUROIMMUN. (3) We applied the manufacturer's recommended cut-off values of <14 RU/mL and <20 RU/mL to define negative aPLA2Rab tests. For the second part of this study, we aimed to confirm the results of the first part through a retrospective analysis that was conducted using our research database. We compared results from aPLA2Rab assays performed on sera that were analyzed by both IFT and ELISA. We included serum samples that were obtained during follow-up, up to 12 months after the start of immunosuppressive therapy.

Summary of three patients with apparent false negative ELISA (ELISA < 2 RU/ml and positive IFT)

Patient 1: Partial remission at the time of ELISA < 2 RU/ml and positive IFT. The IFT remained positive with ELISA < 2 RU/ml, stable kidney function and partial remission throughout 18 months of follow-up.

Patient 2: Partial remission at the time of ELISA < 2 RU/ml and positive IFT. The IFT remained positive and ELISA became > 2 RU/ml with persistent partial remission throughout 28 months of follow-up.

Patient 3: Partial remission at the time of ELISA < 2 RU/ml and positive IFT. The IFT remained positive and ELISA became > 2 RU/ml with a clinical relapse and need for immunosuppressive therapy (cyclophosphamide, rituximab and steroids) 18 months after ELISA < 2 RU/ml. Two months after the start of immunosuppressive therapy ELISA became < 2 RU/ml with negative IFT and partial remission.
